# Precision mapping of schistosomiasis and soil-transmitted helminthiasis among school age children at the coastal region, Kenya

**DOI:** 10.1371/journal.pntd.0011043

**Published:** 2023-01-05

**Authors:** Stella Kepha, Duncan Ochol, Florence Wakesho, Wyckliff Omondi, Sammy M. Njenga, Kariuki Njaanake, Jimmy Kihara, Stephen Mwatha, Chrisistosom Kanyi, Joseph Otieno Oloo, Paul Kibati, Elodie Yard, Laura J. Appleby, Kevin McRae-McKee, Maurice R. Odiere, Sultani Hadley Matendechero

**Affiliations:** 1 Ministry of Health, Division of Vector Borne and Neglected Tropical Diseases, Nairobi, Kenya; 2 Oriole Global Health, Milton-Under-Wychwood, Great Britain; 3 Kenya Medical Research Institute, Nairobi, Kenya; 4 Crown Agents, Great Britain; 5 University of Nairobi, Nairobi, Kenya; 6 Kenya Medical Research Institute, Kisumu, Kenya; Federal University of Agriculture Abeokuta, NIGERIA

## Abstract

**Background:**

Accurate mapping of schistosomiasis (SCH) and soil transmitted helminths (STH) is a prerequisite for effective implementation of the control and elimination interventions. A precision mapping protocol was developed and implemented in the coastal region of Kenya by applying the current World Health Organization (WHO) mapping guide at a much lower administrative level (ward).

**Methods:**

A two-stage cluster survey design was undertaken, with 5 villages in each ward selected. From within each village 50 households were randomly selected, and a single child between the ages of 8 and 14 sampled following appropriate assent. The prevalence and intensity of infection of *Schistosoma mansoni* and STH were determined using the Kato-Katz method (single stool, duplicate slides) and urine filtration for *S*. *haematobium*.

**Results:**

Of the 27,850 school age children sampled, 6.9% were infected with at least one *Schistosoma* species, with *S*. *haematobium* being the most common 6.1% (95% CI: 3.1–11.9), and Tana River County having highest prevalence 19.6% (95% CI: 11.6–31.3). Prevalence of any STH infection was 5.8% (95% CI: 3.7–8.9), with Lamu County having the highest prevalence at 11.9% (95% CI: 10.0–14.1). The most prevalent STH species in the region was *Trichuris trichiura* at 3.1% (95% CI: 2.0–4.8). According to the WHO threshold for MDA implementation, 31 wards (in 15 sub-Counties) had a prevalence of ≥10% for SCH and thus qualify for annual MDA of all age groups from 2 years old. On the other hand, using the stricter Kenya BTS MDA threshold of ≥2%, 72 wards (in 17 sub-Counties) qualified for MDA and were targeted for treatment in 2021.

**Conclusions:**

The precision mapping at the ward level demonstrated the variations of schistosomiasis prevalence and endemicity by ward even within the same sub-counties. The data collected will be utilized by the Kenyan Ministry of Health to improve targeting.

## Author summary

Mapping of schistosomiasis (SCH) and soil-transmitted helminths (STH) is a critical step in understanding where at-risk populations live so as to plan and target available resources in order to achieve maximum impact on disease burden. While there has been progress in mapping of SCH across the world, the current mapping design for SCH is limited in addressing the focal nature of SCH in implementation units. As part of the Breaking Transmission Strategy (BTS) developed by the Kenyan Ministry of Health that focuses on elimination of neglected diseases including SCH and STH, an evaluation was conducted to identify the SCH and STH at-risk population to better target control using a precision mapping approach. The findings from this evaluation provided a comprehensive delineation of the distribution and prevalence of SCH and STH that informed the rollout of community-based mass drug administration for control of SCH and STH at the Kenyan coast.

## Introduction

It is estimated that over 240 million people are acutely or chronically infected with one or more of the *Schistosoma* species [1] worldwide, with the bulk of global transmission occurring in sub-Saharan Africa [2]. In Kenya, where both the intestinal (caused by *Schistosoma mansoni)* and urogenital (caused by *Schistosoma haematobium*) forms of schistosomiasis (SCH) occur, approximately nine million people are estimated to be infected and 17.5 million people are at risk of SCH [3,4]. Long term and heavy infections can result in term chronic morbidity, pain, malnutrition, and sometimes mortality as a result of the body’s inflammatory immune response to eggs laid by adult worms [5].

On the other hand, over 1.5 billion people are infected with at least one species of soil-transmitted helminths (STH) globally [6]. STH are parasitic worms that live in the intestines of humans and other animals. The three predominant STH that can infect humans are *Ascaris lumbricoides*, *Trichuris trichiura*, and hookworm species (*Ancylostoma duodenale* and *Necator americanus*). *A*. *lumbricoides* is the most prevalent, with an estimated global burden of 820 million infections [6]. STH are widely distributed in Kenya, occurring mainly in western and coastal Kenya and selected foci in other parts of the country [7]. People become infected with STHs after ingesting eggs from contaminated soil (*A*. *lumbricoides* and *T*. *trichiura*) or after larvae in the soil (hookworms) have broken through the skin.

Schistosomiasis has been targeted for elimination as a public health problem (EPHP) both within the World Health Organization (WHO)’s Roadmap for Neglected Tropical Diseases (NTDs) 2021–2030 [8] and for elimination and control by 2023 in Kenya’s Breaking Transmission Strategy (BTS) [9]. Up until recently, the cornerstone of schistosomiasis control efforts has been mass drug administration (MDA) of preventive chemotherapy (PC) with praziquantel, targeted at school age children (SAC), with the frequency of treatment determined at the level of implementation unit (IU) by the disease endemicity in a subset of surveyed schools [10]. The eligibility of the IU, usually a district (sub-county in the Kenyan context, and hereafter referred to as such), for PC is determined by the mean prevalence that allows the entire district to be classified as non-endemic (0%), low (>0% and <10%), moderate (> = 10% and <50%) or high (≥ 50%) risk. To date, the WHO’s recommended mapping design has been to select, in each district (sub-county), a sub-sample of up to five schools and 50 children per school for parasitological surveys [11]. Kenya has been treating SAC through the National School based Deworming Programme since 2012 with a previous sub-national baseline survey conducted in 2012 [12], and relying on spatial modelling conducted at that time [7]. Due to the focal nature of schistosomiasis, determining prevalence of an entire district (sub-county in Kenya) based on only a few schools can lead to misclassification of infection risk and the subsequent decision to implement MDA or not may result in overtreatment in some areas and, most importantly, undertreatment or no treatment in areas that need treatment the most [13]. Furthermore, the control strategy, only targeting SAC maybe leaving a significant reservoir of infection, and infection associated morbidity, in both older and younger age groups [14–16]. This misclassification, and narrow targeted treatment range, may be contributing to ongoing challenges in achieving control and elimination targets even in areas where there has been long-term treatment of SAC with high coverage [17,18].

The combination of the focal nature of schistosomiasis transmission; limited availability of resources including drugs and funding; and a desire to move to the next stage of control and elimination following multiple rounds of control via MDA, warrants mapping at a more granular level, using administrative units lower than the sub-district (wards in the case of Kenya) as mapping units. Mapping at a more granular level will refine the understanding of disease distribution and lead to the ability to more definitively target MDA and guide program decisions. Indeed, the current WHO 2021–2030 NTD Roadmap underscores continued micro-mapping and targeting of interventions as *Critical action 2* for reaching schistosomiasis targets [8]. One approach for micro-mapping is precision mapping, also known as *granular* or *sub-district* mapping. Precision mapping provides more refined data by grouping multiple sub-districts, where transmission is likely to be similar according to ecological factors, into a mapping unit to the extent of potentially examining all schools or villages within a sub-unit [19]. Such an approach can guide understanding on where at-risk populations live to effectively target available resources and to achieve maximum impact on disease burden. Accurate delineation of prevalence of schistosomiasis at a much lower level is therefore a critical prerequisite to move from morbidity control towards interruption of schistosomiasis transmission as envisaged in the Kenya Breaking Transmission Strategy (BTS) framework [9].

To address the aforementioned challenge with regards to the limitations in the current conventional mapping design for schistosomiasis, the Kenya Ministry of Health (MoH)’s NTD programme conducted precision mapping for schistosomiasis at the Kenyan Coast. ‘P*recision mapping*’ in this context is defined as conducting sampling at a much finer geographical resolution, examining a higher number of purposively selected schools or villages within lower levels administrative units (wards) below each implementation unit (previously district/sub-county) so as to address the variability in prevalence attributable to the focal nature of schistosomiasis. The mapping guidelines also recommend to map schistosomiasis and soil-transmitted helminthiasis together whenever both diseases are co-endemic in the same region. In this regard, STH were included in the precision mapping.

We detail here the methodologies, results and treatment decisions made following the precision-mapping exercise at the Kenyan Coast. Results are considered in the light of both Kenya’s BTS as well as the current WHO guideline on control and elimination of human schistosomiasis [20].

## Methods

### Ethics statement

Ethical approval for the survey was granted by the Ethics and Scientific Review Committee (ERSC) of AMREF Health Africa in Kenya. Permission to collect data was obtained from district/ ward health offices. Parents and participants were provided with information sheets detailing the purpose of the study and what to expect. Written informed consent by parents or guardians and verbal assent by participating children were obtained prior to enrolment in the study.

### COVID-19 risk mitigation during survey implementation and impact on sampling

In July 2020, following restrictions placed on NTD activities earlier in the year, the WHO issued a new advisory allowing for limited resumption of the community-based activities following a recommended COVID-19 risk assessment and with strict adherence to identified COVID-19 mitigation measures. In line with this WHO directive, a risk assessment was conducted prior to commencement of the survey to evaluate whether the planned mapping activity could be conducted taking into consideration the COVID-19 transmission rates and local preparedness to respond to an outbreak at the time [21]. Precautionary measures were identified to decrease the risk of transmission of COVID-19 from the planned mapping activity, and to strengthen the capacity of the health system to manage any residual risk.

### Study setting and site selection

**Fig 1:**
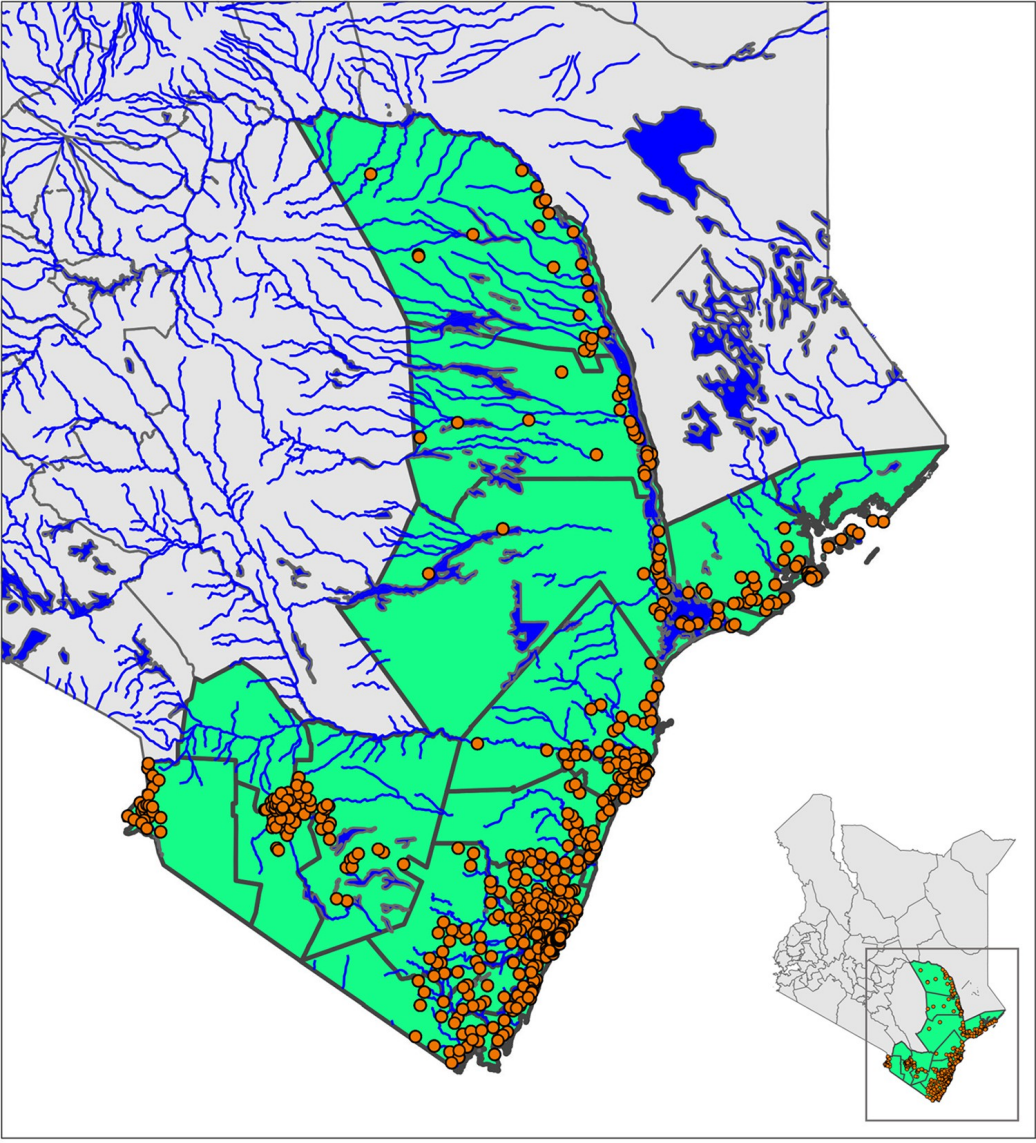
Sample sites/villages. Geographical distribution of samples sites/villages in the coastal region, including bodies of water. Inset: Map of Kenya with sampled counties highlighted. https://data.humdata.org/dataset/cod-ab-ken.

The precision mapping survey was conducted in 6 counties of Mombasa, Kilifi, Kwale, Lamu, Tana River and Taita Taveta on the Kenyan coastal region between October and November 2020. This region is in the Southeastern part of the country (see Fig 1) and covers an area of approximately 79,688 km^2^ with an estimated population of 4,329,474 [22]. The region has a tropical humid climate with an average temperature of about 28°C with a peak of around 39°C. The average rainfall is between 1,000 to 1,100mm per year. This region is known to be endemic for *S*. *haematobium* and STH.

The region has six counties, 26 sub-counties, and 130 wards. Wards are the lowest administrative level, which are ideally demarcated by population numbers, amongst other political considerations, and are thus not all uniform in size. All 130 wards were included in the sample selection. A two-stage cluster survey design was undertaken, whereby, in each ward, five villages (as a proxy for the closed schools) were selected (a total of 650 villages) and 50 school-age children, aged 8–14 years (a minimum of 250 children per ward), were randomly selected from randomly selected households within each village surrounding the selected school. At the time of the survey, schools remained closed in Kenya as a precautionary measure due to the COVID-19 pandemic. Thus while schools formed the sampling unit, school aged children were sampled from the villages that form the catchment for the selected school. This had the added benefit of including any non-enrolled SAC who also are often at greater risk of infection [23].

Selection of villages was guided by previous knowledge of the areas where schistosomiasis transmission is known, suspected, or more likely (within 2km of water bodies, lakes, streams, dams, and irrigation areas). Identification of water bodies was aided by suitability maps based on the available spatial data. Previous knowledge was collected in a step-by-step sequenced manner through desk reviews, analysis of satellite data from MoH, and review of data from the WHO-AFRO’s Expanded Special Project for Elimination of Neglected Diseases Tropical (ESPEN) and from the National School Based Deworming programme (NSBDP). This was then ratified and refined with local knowledge from the Division of Vector Borne and Neglected Tropical Diseases (DVBNTD) laboratories and data from peripheral health facilities. Local public health officials provided lists of potential schools and villages for inclusion. School selection considered the geographical distribution of schools, prioritizing those in close proximity to a water body (those within the 500m were selected first and then working to greater distances up to 2km) in order to be representative in each ward. The survey was conducted in the village were the selected school was located; in most cases the selected village provided most of the children who attend the selected school.

### Recruitment and sample collection

The inclusion criteria for participants were (a) ages 8 to 14 years with parental/guardian consent, (b) assent from children (those above 11 years provided written assent) to participate in the study, (c) resident in the study area/village for the last six months, and (d) no reported receipt of deworming medication in the last 6 months.

Advocacy and sensitization activities were conducted from county through to village level. The cascade was as follows; the national team conducted the county advocacy, the county team conducted sub-county advocacy, the sub-county teams conducted the ward advocacy which targeted the ward public health officers (PHO), the ward PHOs conducted community sensitization in the selected villages through meetings with the village elders and community health volunteers (CHVs) in the selected villages, and lastly the village elders and CHVs conducted household sensitization in their respective villages.

In each village, a total of 50 households were randomly selected and a single child between the ages of 8 and 14 years was randomly sampled in each selected household. On the day of the survey the field officers arrived at households and explained to the heads of the households the purpose of the study, requirements for sample collection, and ensured that appropriate assent/consent was provided by the selected children and parents or guardians prior to participation in the survey.

A Water, Sanitation and Hygiene (WASH) and the disability questionnaire was also administered. Data were collected on toilet access, type of toilet, distance to water source, and type of water source. The household water sources were classified as protected/unprotected and toilet classified as improved/unimproved toilet according to the WHO/UNICEF Joint Monitoring Programme for Water Supply, Sanitation and Hygiene (JMP) indicators [24]. Results from the WASH survey will be presented elsewhere.

Each selected child was provided with instructions and materials for collection and return of their stool and urine sample. At the household, demographic information of the participants including age, sex, and village were collected using the Kobo collect digital platform.

### Laboratory procedures

All stool and urine samples were transferred to the laboratory within four hours of collection for processing on the same day. Stool samples were prepared using the Kato-Katz thick smear method [25] based on duplicate slides (41.7mg template) and examined microscopically for the presence and number of *S*. *mansoni* and STH eggs by two independent technicians. For quality assurance, 10% of all the positives and negative thick smears were re-examined by a third laboratory technician blinded of the results of the other two technicians. Infection intensity was calculated and expressed as the number of eggs per gram of stool (EPG). Urine filtration diagnostic technique was used to detect *S*. *haematobium* eggs in 10ml urine [26]. Infection intensity was determined as number of eggs counted per 10ml urine.

### Data entry and statistical analysis

Data were collected via mobile phone using Kobo Collect (Kobo Inc., Cambridge, MA, USA) and directly uploaded to the cloud for later analysis. To minimize opportunity for user error, entries were selected from a drop-down list where possible.

The final sample of school age children included in the analysis was limited to those with complete data for gender and age, with the latter between years 8 and 14 inclusive, as well as least one valid stool or urine sample. Any STH in individuals with a valid stool sample was defined as having at least one positive infection of any STH species. In the case of SCH, where stool and urine sample within school aged children were not always available/valid, classification of “Any SCH” was limited to school age children with both valid stool and urine samples.

Data management and analyses were performed using R statistical software (version 2022.02.2+461). The prevalence of each *Schistosoma* species and STH species and 95% confidence interval (95% CI) at the sub-county and ward level were calculated using binomial regression analysis taking into account clustering by ward and school/village respectively. Further details on the statistical model used to estimate ward and sub-county prevalences can be found in the supplementary material.

**Fig 2:**
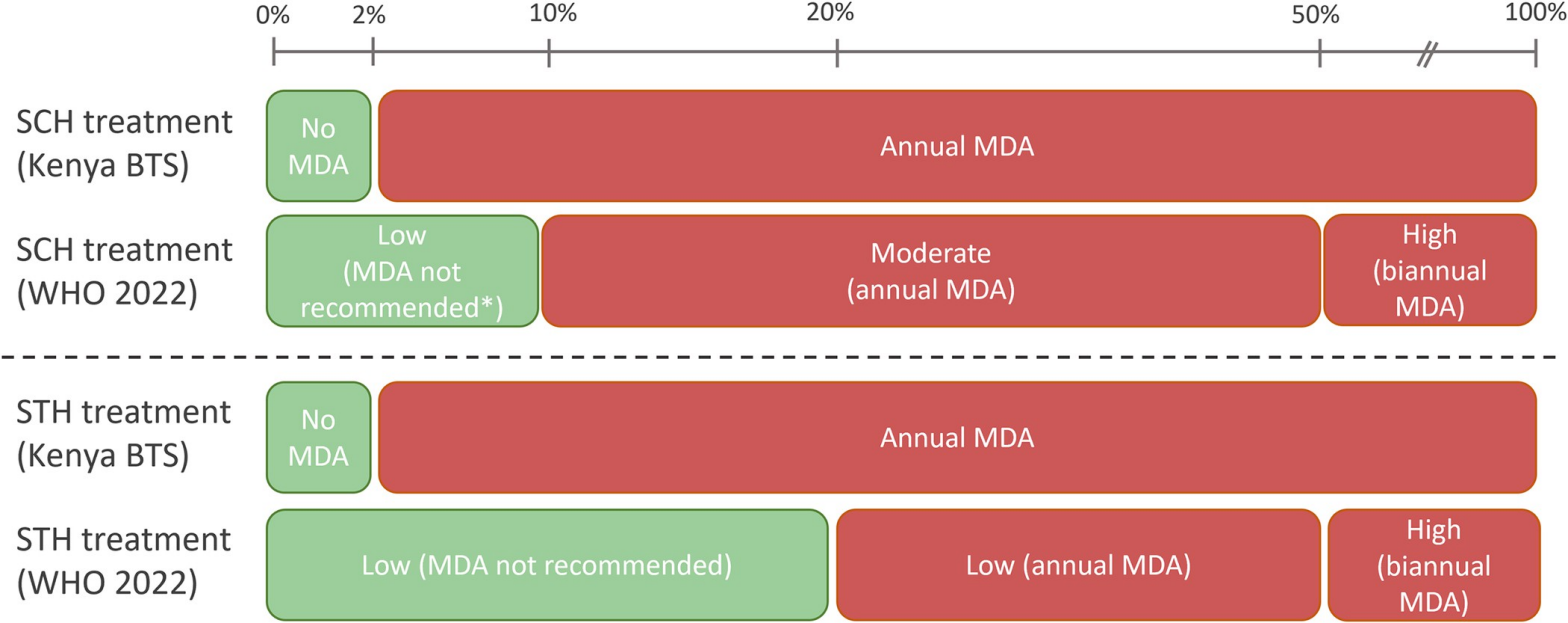
Treatment strategies and WHO recommendations for schistosomiasis and STH control. Red and green sections described the intervals in which MDA is and is not recommended, respectively. WHO = World Health Organization; BTS = Breaking Transmission Strategy; MDA = Mass Drug Administration. * At prevalences of <10% where there has historically been an MDA programme, this is recommended to continue towards the interruption of transmission [27–29].

The updated WHO guidelines identify a prevalence threshold of 10% for annual preventive chemotherapy for schistosomiasis, whereas Kenya’s BTS threshold for initiating MDA is set at 2%. Appreciating that not all areas in the country are at the same stage of control, coupled with the fact that SCH is epidemiologically distinct throughout its geographical distribution [27], the BTS threshold of 2% is considered critical in areas where SCH prevalence is already sufficiently low, with great feasibility to interrupt transmission (i.e. BTS is not rolled out in a “one-size-fits-all” approach). These categories were used to classify villages in the analysis, and presented in such a way that allows the reader to interpret the findings in the context of the varying guidelines and strategies as follows: zero prevalence (0%), low and no MDA (>0% and <2%), low and MDA (Kenya BTS) (≥ 2% and <10%); WHO low (>0 and <10%), WHO moderate (≥10 and <50%) or high prevalence (≥50% prevalence). Similar to SCH, Kenya’s BTS threshold for MDA for STH is ≥ 2%. Therefore, STH was classified into low and no MDA (>0% and <2%) low prevalence and MDA (Kenya BTS) (≥2% and <20%), WHO low risk (≥20% and <50%) and high risk (≥50%). The treatment strategies, cut-offs and recommendations according to Kenya’s BTS and WHO are illustrated in Fig 2 below.

### Map generation

Maps were generated using R statistical software (version 2022.02.2+461 [30] via the package *ggplot2* [31] to plot point prevalence and choropleth maps. Sub-county and ward level shapefiles were imported with support from the MoH whereas site/village geocoordinates were recorded via KoboCollect throughout the data collection process.

### Reprogramming decisions

After data analysis and visualisation, treatment strategies for schistosomiasis were made by the Ministry of Health according to the Kenya BTS (2019–2023), according to categories of >2% or ≤2%. Future praziquantel requirements were calculated based on the updated prevalence thresholds and according to Kenya BTS. Updated drug requirements were then compared to prior requirements.

## Results

A total of 33,254 school age children were recruited across 129 wards and 650 villages. Basuba sub-county, in Lamu county, was not sampled due to ongoing insecurity challenges.

From these, 27,850 school age children had complete demographic (age and gender) data, were between the ages of 8–14 years, inclusive, and provided at least one valid stool or urine sample with successful determination of presence or absence of infection for the two SCH species or STH species, or both. A total of 25,461 school age had complete data for SCH (both stool and urine) whereas 26,512 school age children had complete data for STH (only stool required). The mean age of those in the sample was 11.2 years (SD 1.7) that were split evenly by gender (49.9% females). Further demographic, prevalence, and household information by county is provided in Table 1.

**Table 1:** Study population demographics and prevalence of schistosomiasis and soil-transmitted helminthiasis by county.

	Kilifi	Kwale	Lamu	Mombasa	Taita Taveta	Tana River	Overall
**Number**							
N	7,813	4,372	1,476	6,801	4,358	3,030	27,850
**Sex**							
Male N (%)	3,881 (49.7)	2,179 (49.8)	716 (48.5)	3,472 (51.1)	2,134 (49.0)	1,514 (50.0)	13,954 (50.1)
Female N (%)	3,932 (50.3)	2,193 (50.2)	760 (51.5)	3,329 (48.9)	2,224 (51.0)	1,516 (50.0)	13,896 (49.9)
**Age**							
Mean (SD)	11.2 (1.7)	11.3 (1.7)	11.1 (1.8)	11.2 (1.7)	11.3 (1.7)	10.9 (1.7)	11.2 (1.7)
**SCH prevalence**							
Any SCH % (95% CI)	8.0 (3.5, 17.4)	8.1 (6, 10.9)	7.5 (5.6, 10.0)	1.8 (1.3, 2.7)	2.9 (1.2, 7)	19.6 (11.6, 31.3)	6.9 (3.7, 12.5)
*S*. *mansoni* % (95% CI)	0.3 (0.1, 0.3)	0.2 (0.1, 0.3)	0.3 (0.1, 0.8)	0.4 (0.3, 0.6)	1.5 (0.4, 4.9)	0.4 (0.2, 0.9)	0.5 (0.2, 1.0)
*S*. *haematobium* % (95% CI)	7.4 (3.2, 16.3)	7.5 (5.6, 10.1)	6.9 (4.9, 9.7)	1.4 (0.9, 2.2)	1.4 (0.6, 3.0)	18.3 (11.2, 28.4)	6.1 (3.1, 11.9)
**STH prevalence**							
Any STH % (95% CI)	7.0 (6, 8.3)	9.1 (4.2, 18.8)	11.9 (10, 14.1)	3.6 (2.4, 5.5)	0.8 (0.3, 1.6)	6.8 (2.9, 15.4)	5.8 (3.7, 8.9)
Hookworm % (95% CI)	4.3 (3.3, 5.6)	6.5 (3.0, 13.8)	3.2 (2.4, 4.3)	0.6 (0.3, 1.3)	0.2 (0.0, 0.7)	0.3 (0.2, 0.4)	2.6 (1.1, 6.0)
*A*. *lumbricoides* % (95% CI)	1.0 (0.5, 1.8)	0.7 (0.5, 1.1)	0.1 (0.1, 0.1)	0.4 (0.2, 0.7)	0.1 (0.0, 0.2)	0.6 (0.3, 1.0)	0.6 (0.3, 1.0)
*T*. *trichiura* % (95% CI)	2.3 (0.7, 3.2)	3.2 (1.1, 9.2)	8.8 (7.7, 10.1)	2.8 (2.0, 4.1)	0.5 (0.2, 1.2)	6.1 (2.2, 15.9)	3.1 (2.0, 4.8)

### Schistosomiasis

The overall prevalence of schistosomiasis in the region was 6.9% (95% CI:3.7–12.5). Overall, prevalence was low in Mombasa (1.8% 95%, CI:1.3–2.7) and Taita Taveta counties (2.9%, 95%: CI 1.2–7.0), and highest in Tana River county (19.6%, 95% CI: 11.6–31.3) as shown in Table 1. As expected, the prevalence of *S*. *haematobium* was higher prevalence (6.1%, 95% CI: 3.1–11.9) relative to *S*. *mansoni* (0.5%, 95% CI: 0.2–1.0) in this region. For Kenya’s BTS 72 wards had a prevalence of ≥2% and thus qualify for ongoing annual MDA (Table 2). Infection intensity for schistosomiasis was low; of the infected school age children it was on average 193.1 epg for *S*. *mansoni* with a maximum, and 99.6 eggs per 10ml urine for *S*. *haematobium*. Prevalences of *S*. *mansoni and S*. *haematobium* were combined for the purposes of this analysis.

**Table 2:** Comparison of the number of wards and sub-counties that qualify for MDA according to Kenya’s treatment cut-offs for schistosomiasis.

		No. wards qualifying for treatment when aggregated at sub-county level		
		No MDA	MDA required	Total
No. wards qualifying for treatment when aggregated at ward level (actual treatment requirement)	No *MDA*	33	24	**57**
	MDA required	7	65	**72**
	**Total**	**40**	**89**	**129**

### SCH prevalence by ward and sub-county

Maps of schistosomiasis prevalence were developed in such a way as to reflect both the WHO MDA criteria [29], as well as Kenya’s BTS threshold for MDA, and to allow readers to interpret the maps in the desired manner. Maps were plotted by ward (data can be found in S1 Table and includes prevalence estimates at the sub-county level), as well as by village, and illustrate the range of infection prevalence’s in the region.

**Fig 3:**
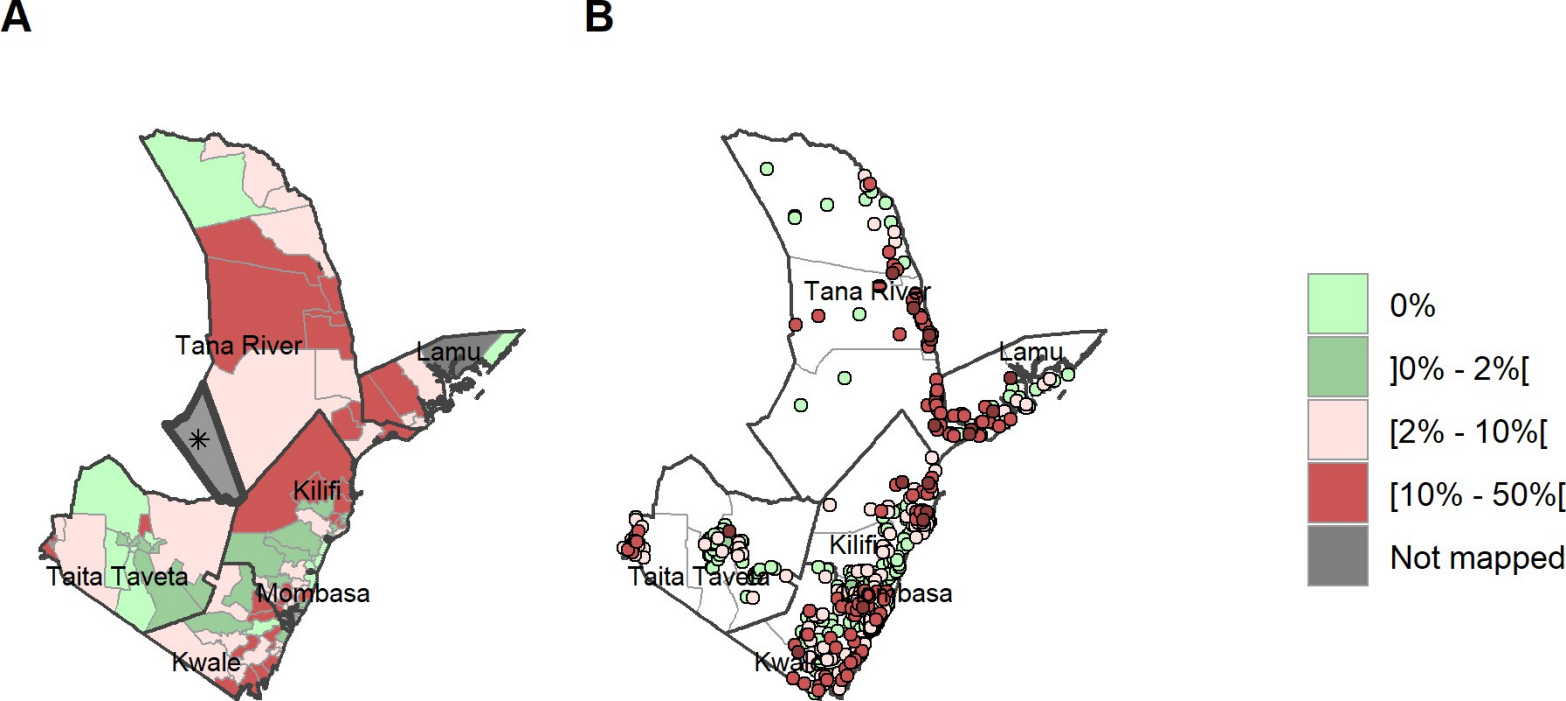
Any SCH prevalence by ward and site/village. (A) Prevalence of any SCH by ward. (B) Prevalence of any SCH by site/village. Maps include county boundaries and labels. Busaba ward (Lamu county) was not mapped. *the Tasvo East National Park region is administratively part of Garsen South, but was not mapped. (https://data.humdata.org/dataset/cod-ab-ken).

Fig 3 illustrates the prevalence of SCH by ward (A) and according to village level point prevalence (B). The ward level map illustrates the combined prevalences showing where the non-endemic wards are located, and where treatment is required based on the 2% treatment threshold, as well as WHO preventive chemotherapy recommendation.

The point prevalence map of infection at village level (Fig 3B) clearly shows the high focality of schistosomiasis prevalence within the region, as well as significant variations in prevalence of schistosomiasis at ward level and within sub-counties. Infection prevalence over 10% for any SCH was recorded in Kilifi, Kwale, Lamu and Tana River, which has the highest point prevalence of 47.5% (supplementary material). These high prevalence points, when combined with other villages at ward level, are translated into areas of moderate infection prevalence at the ward level (Fig 3A).

### Soil transmitted helminthiasis (STH)

The overall prevalence of any STH infection at county level was low for all three STH species, as shown in Table 1. The overall prevalence of any STH infection in the region was 5.8% (95% CI:3.7–8.9), with the highest prevalence at county level reported in Lamu county at 11.9% (95% CI:10.0–14.1), and the lowest in Taita Taveta county at 0.8% (95% CI:0.3–1.6). The most prevalent STH species in the region was *T*. *trichiura* at 3.1% (95% CI:2.0–4.8), followed by hookworm at 2.6%, (95% CI: 1.1–6.0) and *A*. *lumbricoides* (0.6%, 95% CI:0.3–1.0). Lamu and Tana River counties had the highest prevalences of *T*. *trichiura* infection (8.8% and 6.1%, respectively), while Kwale and Kilifi counties had the highest prevalences of hookworm (6.5% and 4.3%, respectively). *A*. *lumbricoides* infection was low in all counties, with the highest prevalence in Kilifi at 1.0%. Infection prevalence at county level for all STH species remained below the WHO 20% cut-off for MDA.

### STH prevalence by ward and sub-county and species

**Fig 4:**
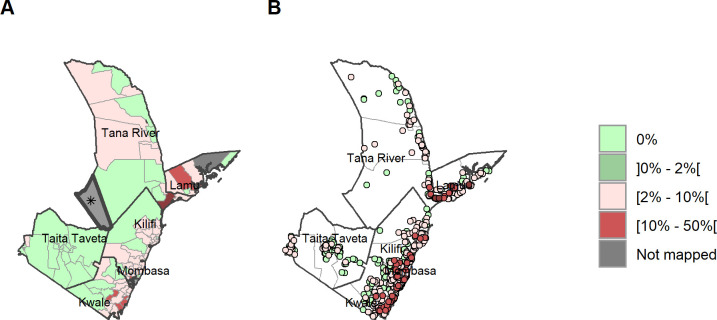
Any STH prevalence by ward and site/village. (A) Prevalence of any STH by ward. (B) Prevalence of any STH by site/village. Maps include county boundaries and labels. Busaba ward (Lamu county) was not mapped. *the Tasvo East National Park region is administratively part of Garsen South, but was not mapped. (https://data.humdata.org/dataset/cod-ab-ken).

**Fig 5:**
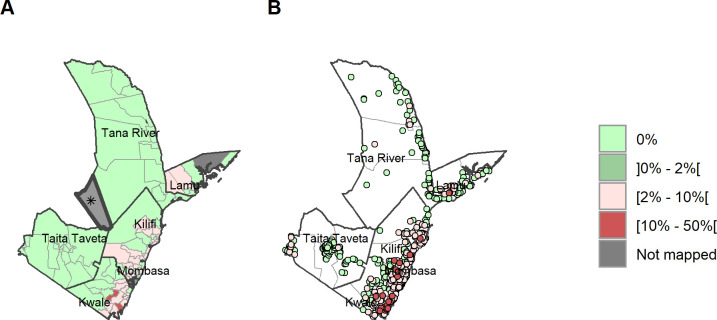
Hookworm prevalence by ward and site/village. (A) Prevalence of hookworm by ward. (B) Prevalence of hookworm by site/village. Maps include county boundaries and labels. Busaba ward (Lamu county) was not mapped. *the Tasvo East National Park region is administratively part of Garsen South, but was not mapped. (https://data.humdata.org/dataset/cod-ab-ken).

**Fig 6:**
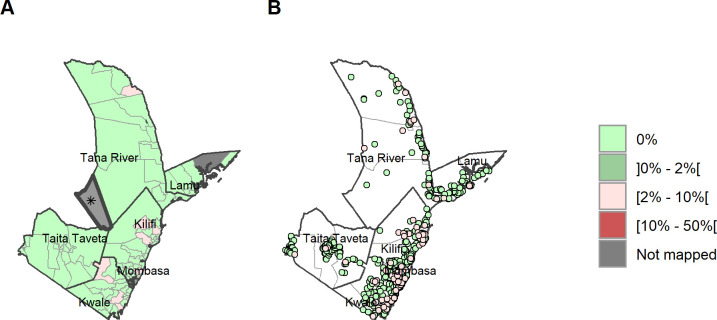
*Ascaris lumbricoides* prevalence by ward and site/village. (A) Prevalence of *A*. *lumbricoides* by ward. (B) Prevalence of *A*. *lumbricoides* by site/village. Maps include county boundaries and labels. Busaba ward (Lamu county) was not mapped. *the Tasvo East National Park region is administratively part of Garsen South, but was not mapped. (https://data.humdata.org/dataset/cod-ab-ken).

**Fig 7:**
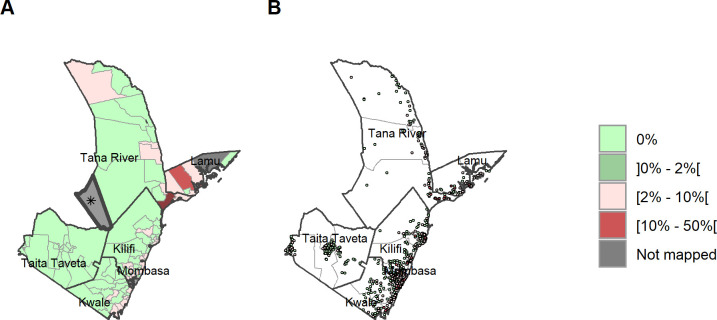
*Trichuris trichiura* prevalence by ward and site/village. (A) Prevalence of *T*. *trichiura* by ward. (B) Prevalence of *T*. *trichiura* by site/village. Maps include county boundaries and labels. Basuba ward (Lamu county) was not mapped. *the Tasvo East National Park region is administratively part of Garsen South, but was not mapped (https://data.humdata.org/dataset/cod-ab-ken).

A total of 121 wards had a prevalence of <20% and thus do not qualify for MDA according to WHO criteria, 14 of those 120 wards were non-endemic (0% prevalence) for any STH (Fig 4) across all 6 counties (S1 and S2 Tables). At ward level, *A*. *lumbricoides* infection remained low and below 5% in all wards (Fig 6A). Hookworm had a maximum prevalence of 26.5% (Fig 5A) with the highest prevalences in Kwale county. *T*. *trichiura* infection had a maximum ward-level prevalence of 50.4% found in Tana River (Fig 7A). One ward presented with infection prevalence for any STH of over 50% in Tana River (51.9% in Kipini West ward in Garsen sub-county). Mombasa and Taita Taveta counties did not have any wards with STH prevalence above 20%.

### Reclassification of sub-counties for schistosomiasis treatment according to precision mapping results

**Fig 8:**
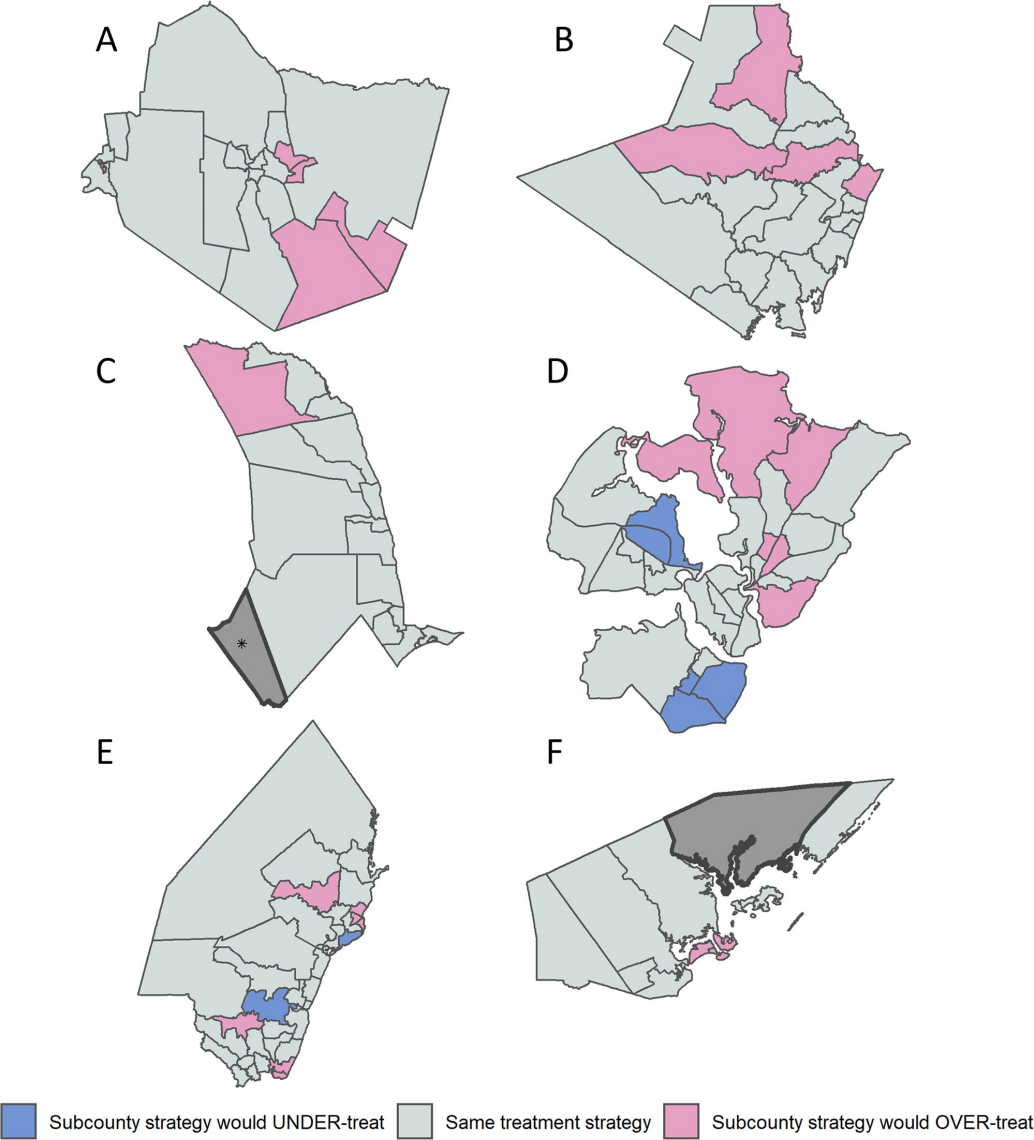
Comparison of Sub-county and Ward strategies by county. (A) Taita Taveta county. (B) Kwale county. (C) Tana River county. (D) Mombasa county. (E) Kilifi county. (F) Lamu county. Overtreatment and undertreatment when designating treatment according to sub-county versus ward. Busaba ward (Lamu county) was not mapped. *the Tasvo East National Park region is administratively part of Garsen South, but was not mapped. (https://data.humdata.org/dataset/cod-ab-ken).

*Note*: *treatment cut-off is based on a* ≥2% prevalence threshold.

Using these updated prevalence maps, the Kenyan MoH’s NTD program aims to reclassify treatment at the ward level, to target breaking transmission, defined in BTS as <2% prevalence. A total of 72 out of the 129 wards had a prevalence ≥2% and will continue to be targeted to receive annual MDA for schistosomiasis at the community level, including the expanded target population of adults inclusive of women of reproductive age (WRA). Table 2, together with Fig 8, illustrates the distribution of prevalence categories for schistosomiasis according to Kenya’s treatment categories for BTS when disaggregated at ward level as well as sub-county level. When determining treatment on a ward level, 72 wards out of the total 129 wards have a treatment prevalence of ≥2% thus qualifying for annual treatment. In contrast, determining the MDA based on prevalence by sub-county, 17 sub-counties, (comprising of 89 wards) had an average prevalence of ≥2%, but contained within these sub-counties are 24 wards which actually have a prevalence of <2% and which would have been overtreated. Similarly at the sub-county level, there would have been 7 wards mis-categorized to not receive treatment despite them having a prevalence ≥2%, leading to undertreatment in these wards (Table 2 and Fig 8).

#### Refined resource requirements

The updated treatment strategy, using wards as opposed to sub-county designations, has led to a decrease in total number of targeted wards, a decrease in the total population requiring treatment, and a decrease in the praziquantel requirements, as shown in Table 3 below.

**Table 3:** Number of wards, population targeted and praziquantel requirements in the region prior to and post-granular mapping.

	Sub-county designation			ward level designation		
County	No wards targeted	SAC	Adults	No of wards targeted	SAC	Adults
Kilifi	24	293,410	677,955	19	219,410	506,969
Kwale	20	255,911	591,309	16	209,488	484,044
Lamu	7	42,500	98,201	5	29,223	67,523
Mombassa	12	149,111	344,537	12	188,338	435,174
Taita-Taveta	11	59,048	136,436	6	25,924	59,901
Tana River	15	90,535	208,770	14	84,800	195,939
Total	89	890,515	2,057,208	72	757,183	1,749,550
Praziquantel estimate		2,226,288	7,200,228		1,892,958	6,123,425

*Abbreviations*: *SAC*, *School-age children; PZQ*, *praziquantel;*. *NB* Praziquantel requirements are estimated as 2.5 tablets per SAC; 3.5 tablets per adult and WRA.

## Discussion

In Kenya, the MoH and Ministry of Education (MoE) have been conducting a school-based deworming programme (NSBDP) since 2012, targeting all SAC. In reflection of a decade of school-based deworming, and to more effectively target treatment to achieve Kenya’s ambitious elimination goals, the MoH aimed to reassess the prevalence of schistosome infection in 6 counties in the coastal region on a more granular scale. Precision mapping was undertaken between October and November of 2020 in 6 counties in the coastal region of Kenya. The mapping has generated useful data at a finer resolution for SCH and STH that has supported updating of prevalence maps that will guide future treatment strategies, helping determine where MDA is no longer required or requires a different strategy in order to break transmission in line with Kenya’ BTS [9].

Whilst direct comparisons are difficult to be made due to the differences in sampling methodology, overall, the prevalences of both STH and *Schistosoma spp*. Notably, there have been minor increases in prevalences in the region since the survey was conducted in 2017 [17]. For schistosome infections, this is likely due to the purposive nature of the sampling strategy employed during this survey, focussing on villages near to likely infection areas and water sources.

The conventional WHO mapping guidelines aggregates prevalences at the sub-county (district) whereas this precision mapping was done at the ward level in a reflection of both the focal nature of schistosomiasis transmission, and Kenya’s target of elimination of schistosomiasis [9]. Following the survey, wards were classified based on their need for treatment intervention as: no MDA required (<2%), or requiring MDA (≥2%) for the purposes of making treatment decisions in accordance with Kenya’s BTS [32]. In areas which do not qualify for MDAs, the MoH will promote routine surveillance to continue monitoring the trends and to ensure the low prevalence is sustained. In addition, for all areas (both under MDA and those without), the MoH will promote WASH activities and will ensure PZQ is available in health facilities to treat individual cases.

The use of this precision mapping methodology has highlighted areas of both high and continuing schistosome infection, as well as no infection even within the same sub-counties. These areas would have been at risk of over- or under-treatment utilizing standard mapping techniques, but are now identified for targeted treatment. Using this new data, Kenya has now updated its prevalence maps, and MDA will be conducted on a ward level (as the IU) as opposed to a sub-county level, allowing for a greater degree of precision and improved allocation of resources.

The prevalence and distribution of SCH and STH infections in this survey has several implications for mass treatment programs. As a result of this mapping, a total of 72 wards with a prevalence of ≥2% will require annual treatment targeting all SAC and adults, including WRA in line with the WHO guidelines [20]. Conversely, the results of the mapping have identified 57 wards which no longer require MDA, inclusive of 24 wards which may have been overtreated if following a sub-county level designation rather than ward level. This alone will save an estimated 1,410,133 tablets of PZQ which can now be reassigned to areas in need.

Overall, the prevalence of STH was found to be <2% in the majority of wards with only pockets of high prevalence in Tana River, Lamu and Kwale. *Trichuris* was the most prevalent STH species, followed by hookworm. There was little *A*. *lumbricoides* infection found. These results may be a reflection of the impact of the 10 years of school based deworming in the area, and the efficacy of albendazole and mebendazole against *A*. *lumbricoides* infection, in comparison to, in particular *T*. *trichiura* infection [33]. In the future, treatment strategies could be indicated based on species-specific prevalences, with albendazole treatment focussed on areas where hookworm is more prevalent, and mebendazole where *T*. *trichiura* is predominant. The dynamics of hookworm infection in particular, mean that adults in the community will benefit from Kenya’s plans to expand treatment to the wider community. Based on the findings in the present survey, 50 wards would require co-administration of PZQ and albendazole or mebendazole, whereas 22 wards would require PZQ alone and 28 wards albendazole or mebendazole alone.

The results shown here illustrate that sub-county, or district, prevalences do not equate to ward level prevalences, leading to challenges if utilizing the sub-county rather than ward to designate schistosomiasis prevalence. Utilizing appropriate geographical sampling and ecological zoning is therefore critical when undertaking prevalence mapping for a highly focal infection. Precision mapping allows for identifying pockets of schistosome infection, avoids diluting areas of high or low transmission caused by aggregating prevalences, and prevents missing treatment opportunities. For example, this mapping exercise has identified infection pockets in Taita Taveta whereas a previous survey indicated zero endemicity [17].

Developing a further understanding of reasons behind the high prevalences in wards which have received multiple rounds of preventive chemotherapy will be critical in designing locally tailored interventions and targeting an effective and comprehensive control and elimination strategy in these areas, including supportive WASH and environmental interventions. Importantly, identifying any pockets of high prevalence infection, and potential hotspots that maybe resistant to treatment will also be critical [34].

Following this mapping, any ward with prevalence ≥2% will continue with treatment in accordance with the BTS. In Kenya, the precision mapping implemented at the coast directly informed the SCH MDA conducted in July and September 2022, funded by the ARISE-NTDs programme. In addition, for both STH and SCH, the MoH has now incorporated additional delivery platforms such as community-based platform in order to effectively expand treatment to out-of-school children; school age children; and adults. Drugs for SAC will continue to be procured from the WHO via the global donation and distributed with support from the ARISE-NTDs programme. Additional praziquantel requirements for the wider community will be fulfilled by a separate donation from Merck with additional support for procurement by donor deworming programmes. Pre-SAC will be included in the targets once a paediatric formulation becomes widely available for 2–5 year olds. Recent efforts by WHO to update the Joint Application Package (JAP) for NTDs to allow countries to report prevalences and place medicine requests based on administrative units below the district implementation unit further enhances the operationalism and implementation of precision mapping efforts for many endemic countries. Whereas the 2% threshold may seem too stringent/overambitious, this goes to the heart of the strategic objective for the program as outlined in the Kenya BTS. Indeed, examples exist around the globe where lower thresholds were effected in line with strategic objectives. For instance, in endemic areas of China, where the strategy is to control and eventually interrupt transmission of *S*. *japonicum*, all people aged 5–65 years are treated, regardless of community infection status [35]. In Egypt, the mass distribution of PZQ evolved over time where mass treatment in 1997 was given in all communities with ≥ 20% SAC prevalence, but this SAC threshold gradually decreased to ≥ 10% in 1999, ≥ 5% in 2000, ≥ 3.5% in 2002, and ≥ 3% in 2003 [36].

Overall resource savings may be significant when considering the costs of transport, training, drug procurement, social mobilization, monitoring and staff time at all levels of the health service. For Kenya, further investigation will reveal the extent of these resource savings over the short term, as the programme adjusts with reduced geographical coverage, but increased targets; and over the long term, with anticipated expedited elimination of transmission due to a more focussed control programme.

### Limitations of the study

Due to the nature of the survey which was cross-sectional, and the fact that only a single stool and urine sample was collected, there is a chance that the true prevalence in the wards may have been underestimated. Application of this purposive sampling methodology requires in-depth understanding and knowledge of the geography and cultural context, in order to appropriately and thoroughly select representative areas appropriate for sampling. Additionally, results are limited to those administrative areas which were sampled; extrapolation to other areas should be done with caution. Nonetheless, application of the precision/granular mapping approach to a focally distributed disease, results in a more accurate prevalence estimate to lower administrative units at-risk of disease. This approach will reduce overtreatment and resource wastage, and in particular, free up limited supplies of PZQ for distribution to those in need. Critically, the fact that the methodology minimizes undertreatment of schistosomiasis, a chronic and debilitating disease when left untreated, means it should be expanded to other geographies with urgency. This approach has been used in other countries with successful results, and will support progress towards the WHO 2030 schistosomiasis elimination goals.

## Conclusion

The precision mapping at the ward level demonstrated the variations of schistosomiasis prevalence and endemicity even within the same sub-counties and wards. It has provided a finer delineation of pockets for SCH based on the wards within the sub-county. The Kenyan MoH is currently using this data to improve targeting and resource allocation in order to progress towards breaking transmission in the coastal area.

Prevalence maps will be distributed to local levels for ownership, planning and decision making in an effort to focus on hot-spots and higher transmission areas. The results will contribute towards the efforts of breaking transmission of schistosomiasis in Kenya and precision mapping methodology should be replicated in other regions of the Country.

## Supporting information

S1 Fig Description of the model used calculating for ward and sub-county prevalence. (DOCX)

S1 Table Schistosomiasis and soil-transmitted helminthiasis prevalence by sub-county. (DOCX)

S2 Table Schistosomiasis and soil-transmitted helminthiasis prevalence by ward. (DOCX)
