# Supplementary material for: Precision mapping of schistosomiasis and soil-transmitted helminthiasis among school age children at the coastal region, Kenya
Source: PLoS Negl Trop Dis. 2023 Jan 5;17(1):e0011043. doi: 10.1371/journal.pntd.0011043 (PMC9847902; doi:10.1371/journal.pntd.0011043)
Supplement: S1 Fig — (DOCX) [file pntd.0011043.s001.docx]

Supplementary material

**Description of the model used calculating for ward and sub-county prevalence**

**We provide the model used for calculating ward and sub-county prevalence estimates below:**

$$\mathbf{ln}\left( \frac{\boldsymbol{p}_{\boldsymbol{ij}}}{\boldsymbol{1-}\boldsymbol{p}_{\boldsymbol{ij}}} \right)\boldsymbol{=}\boldsymbol{\beta}_{\boldsymbol{0}\boldsymbol{j}}\boldsymbol{+}\boldsymbol{\beta}_{\boldsymbol{1}}\boldsymbol{X}_{\boldsymbol{ij}\boldsymbol{1}}\boldsymbol{+\ldots+}\boldsymbol{\beta}_{\boldsymbol{k}}\boldsymbol{X}_{\boldsymbol{ijk}}\boldsymbol{+}\boldsymbol{v}_{\boldsymbol{ij}}$$

$$\boldsymbol{\beta}_{\boldsymbol{0}\boldsymbol{j}}\boldsymbol{=}\boldsymbol{\beta}_{\boldsymbol{0}}\boldsymbol{+}\boldsymbol{u}_{\boldsymbol{0}\boldsymbol{j}}$$

$$\boldsymbol{u}_{\boldsymbol{0}\boldsymbol{j}}\boldsymbol{=N}\left( \boldsymbol{0,}\boldsymbol{\sigma}_{\boldsymbol{u}\boldsymbol{0}}^{\boldsymbol{2}} \right)$$

$$\boldsymbol{v}_{\boldsymbol{1}\boldsymbol{j}}\boldsymbol{=N}\left( \boldsymbol{0,}\boldsymbol{\sigma}_{\boldsymbol{v}\boldsymbol{0}}^{\boldsymbol{2}} \right)$$

**Where** $\boldsymbol{p}_{\boldsymbol{ij}}$ **is the probability that individual** $\boldsymbol{i}$ **in cluster** $\boldsymbol{j}$ **is infected.** $\boldsymbol{\beta}_{\boldsymbol{0}}$ **is the mean intercept and** $\boldsymbol{\beta}_{\boldsymbol{k}}$ **is the log odds ratio associated with a one unit increase in variable** $\boldsymbol{X}_{\boldsymbol{k}}$**.** $\boldsymbol{u}_{\boldsymbol{0}\boldsymbol{j}}\boldsymbol{\sim N(0,}\boldsymbol{\sigma}_{\boldsymbol{u}\boldsymbol{0}}^{\boldsymbol{2}}\boldsymbol{)}$ **represents a specific deviance from** $\boldsymbol{\beta}_{\boldsymbol{0}}$ **for individual** $\boldsymbol{i}$ **which accounts for the variability in the likelihood of infection between clusters.** $\boldsymbol{v}_{\boldsymbol{ij}}\boldsymbol{\sim N(0,}\boldsymbol{\sigma}_{\boldsymbol{v}\boldsymbol{0}}^{\boldsymbol{2}}\boldsymbol{)}$ **represents the random error which accounts for the variability between individuals.**
