# Supplementary material for: Precision mapping of schistosomiasis and soil-transmitted helminthiasis among school age children at the coastal region, Kenya
Source: PLoS Negl Trop Dis. 2023 Jan 5;17(1):e0011043. doi: 10.1371/journal.pntd.0011043 (PMC9847902; doi:10.1371/journal.pntd.0011043)
Supplement: S2 Table — (DOCX) [file pntd.0011043.s003.docx]

*Supp Table 2: schistosomiasis and soil-transmitted helminthiasis prevalence by ward*

| County | Ward | Any SCH | | Any STH | |
| --- | --- | --- | --- | --- | --- |
|  |  | Prevalence  (95% CI) | Prevalence grouped | Prevalence  (95% CI) | Prevalence grouped |
| Kilifi | Adu | 10.8 (4.7, 22.9) | [10%, 50%[ | 1.88 (0.5, 6.7) | <2% |
| Kilifi | Bamba | 1.07 (0.4, 3.2) | <2% | 6.95 (1.3, 29.7) | [2%, 20%[ |
| Kilifi | Chasimba | 2.42 (1.4, 4.1) | [2%, 10%[ | 4.78 (1.7, 12.6) | [2%, 20%[ |
| Kilifi | Dabaso | 1.04 (0.3, 3.3) | <2% | 9.45 (5.5, 15.7) | [2%, 20%[ |
| Kilifi | Ganda | 22.27 (6.8, 53.1) | [10%, 50%[ | 15.12 (10.8, 20.8) | [2%, 20%[ |
| Kilifi | Ganze | 1.79 (0.8, 4) | <2% | 6.67 (2.6, 16.3) | [2%, 20%[ |
| Kilifi | Garashi | 1.95 (0.9, 4.4) | <2% | 10.19 (3.6, 25.7) | [2%, 20%[ |
| Kilifi | Gongoni | 37.3 (17.8, 62) | [10%, 50%[ | 3.56 (1.9, 6.4) | [2%, 20%[ |
| Kilifi | Jaribuni | 3.13 (1.1, 8.6) | [2%, 10%[ | 7.73 (3.4, 16.7) | [2%, 20%[ |
| Kilifi | Jilore | 5.56 (2.7, 11.1) | [2%, 10%[ | 4.61 (3.4, 6.3) | [2%, 20%[ |
| Kilifi | Junju | 14.75 (6.7, 29.5) | [10%, 50%[ | 13 (5.7, 27.1) | [2%, 20%[ |
| Kilifi | Kakuyuni | 10 (2.5, 32.8) | [10%, 50%[ | 2.71 (1, 7.1) | [2%, 20%[ |
| Kilifi | Kaloleni | 14.04 (6.8, 26.8) | [10%, 50%[ | 17.86 (10.4, 29) | [2%, 20%[ |
| Kilifi | Kambe | 6.93 (3.2, 14.3) | [2%, 10%[ | 4.81 (2.3, 9.9) | [2%, 20%[ |
| Kilifi | Kayafungo | 3.72 (1.7, 8.1) | [2%, 10%[ | 0 (0, 0) | <2% |
| Kilifi | Kibarani | 0 (0, 0) | <2% | 12.79 (8.1, 19.5) | [2%, 20%[ |
| Kilifi | Magarini | 38.64 (30.2, 47.8) | [10%, 50%[ | 9.37 (6.7, 12.9) | [2%, 20%[ |
| Kilifi | Malindi Town | 0.55 (0.1, 3) | <2% | 0.54 (0.1, 2.8) | <2% |
| Kilifi | Marafa | 17.96 (6.8, 39.7) | [10%, 50%[ | 5.95 (2.9, 12) | [2%, 20%[ |
| Kilifi | Mariakani | 4.09 (2.8, 6) | [2%, 10%[ | 6.82 (2.5, 17.2) | [2%, 20%[ |
| Kilifi | Matsangoni | 0 (0, 0) | <2% | 5.06 (2.8, 9.1) | [2%, 20%[ |
| Kilifi | Mnarani | 0 (0, 0) | <2% | 7.26 (2.1, 22.2) | [2%, 20%[ |
| Kilifi | Mtepeni | 0.87 (0.3, 2.8) | <2% | 1.72 (0.8, 3.7) | <2% |
| Kilifi | Mwanamwinga | 0.41 (0.1, 2.2) | <2% | 8.68 (2.9, 23.2) | [2%, 20%[ |
| Kilifi | Mwarakaya | 4.52 (1.8, 11) | [2%, 10%[ | 4.92 (3, 8) | [2%, 20%[ |
| Kilifi | Mwawesa | 8.85 (2.4, 27.7) | [2%, 10%[ | 24.38 (18.2, 31.9) | [20%, 50%[ |
| Kilifi | Rabai | 22.37 (10.6, 41.3) | [10%, 50%[ | 8.85 (5.7, 13.6) | [2%, 20%[ |
| Kilifi | Ruruma | 10.91 (5.9, 19.2) | [10%, 50%[ | 6.63 (2.5, 16.4) | [2%, 20%[ |
| Kilifi | Sabaki | 1.89 (0.8, 4.4) | <2% | 4.17 (2.2, 7.9) | [2%, 20%[ |
| Kilifi | Shella | 1.06 (0.2, 5.9) | <2% | 7.29 (2.5, 19.5) | [2%, 20%[ |
| Kilifi | Shimo La Tewa | 1.54 (0.2, 9.9) | <2% | 11.94 (4.6, 27.5) | [2%, 20%[ |
| Kilifi | Sokoke | 1.31 (0.7, 2.6) | <2% | 1.22 (0.4, 3.9) | <2% |
| Kilifi | Sokoni | 0 (0, 0) | <2% | 1 (0.3, 2.9) | <2% |
| Kilifi | Tezo | 0 (0, 0) | <2% | 8.56 (4.1, 17.1) | [2%, 20%[ |
| Kilifi | Watamu | 2.6 (1.1, 6.1) | [2%, 10%[ | 6.91 (3.3, 13.8) | [2%, 20%[ |
| Kwale | Chengoni Samburu | 1.44 (0.5, 4.5) | <2% | 0 (0, 0) | <2% |
| Kwale | Dzombo | 2.9 (0.8, 10.5) | [2%, 10%[ | 6.05 (3, 11.8) | [2%, 20%[ |
| Kwale | Gombato/bongwe | 3.94 (1.9, 8.1) | [2%, 10%[ | 21.34 (10.5, 38.5) | [20%, 50%[ |
| Kwale | Kasemeni | 11.01 (4.8, 23.4) | [10%, 50%[ | 6.88 (3.2, 14.3) | [2%, 20%[ |
| Kwale | Kinango | 0 (0, 0) | <2% | 2.71 (1.2, 6.1) | [2%, 20%[ |
| Kwale | Kinondo | 15.72 (9.2, 25.5) | [10%, 50%[ | 25 (20.1, 30.7) | [20%, 50%[ |
| Kwale | Kubo South | 4.22 (2.3, 7.6) | [2%, 10%[ | 7.23 (4.8, 10.8) | [2%, 20%[ |
| Kwale | Mackinon | 6.05 (2.9, 12.1) | [2%, 10%[ | 5.45 (3.8, 7.8) | [2%, 20%[ |
| Kwale | Mkongani | 11.7 (5.1, 24.4) | [10%, 50%[ | 23.16 (12.4, 39) | [20%, 50%[ |
| Kwale | Mwavumbo | 12.04 (7.7, 18.3) | [10%, 50%[ | 0.46 (0.1, 2.5) | <2% |
| Kwale | Mwereni | 8.79 (4.7, 15.8) | [2%, 10%[ | 0.41 (0.1, 2.3) | <2% |
| Kwale | Ndavaya | 3.87 (1.4, 10) | [2%, 10%[ | 1.27 (0.2, 6.8) | <2% |
| Kwale | Pongwekikoneni | 10 (6.5, 15.2) | [10%, 50%[ | 17.94 (9.6, 31.1) | [2%, 20%[ |
| Kwale | Puma | 0.54 (0.1, 3.3) | <2% | 0.53 (0.1, 2.8) | <2% |
| Kwale | Ramisi | 16.11 (9, 27.1) | [10%, 50%[ | 36.28 (33.7, 38.9) | [20%, 50%[ |
| Kwale | Tiwi | 4.5 (2.1, 9.2) | [2%, 10%[ | 5.31 (3.2, 8.8) | [2%, 20%[ |
| Kwale | Tsimba Golini | 26.27 (11.9, 48.4) | [10%, 50%[ | 4.72 (2.6, 8.5) | [2%, 20%[ |
| Kwale | Ukunda | 5.98 (3.1, 11.1) | [2%, 10%[ | 12.11 (8.3, 17.4) | [2%, 20%[ |
| Kwale | Vanga | 12.12 (6.7, 20.9) | [10%, 50%[ | 8.15 (5.6, 11.8) | [2%, 20%[ |
| Kwale | Waa | 0.49 (0.1, 2.7) | <2% | 6.22 (1.6, 21.1) | [2%, 20%[ |
| Lamu | Bahari | 7.17 (3.2, 15.4) | [2%, 10%[ | 3.69 (2, 6.8) | [2%, 20%[ |
| Lamu | Faza | 1.28 (0.5, 3.6) | <2% | 5.95 (4.1, 8.6) | [2%, 20%[ |
| Lamu | Hindi | 4.95 (0.9, 22.1) | [2%, 10%[ | 5.5 (2.7, 11) | [2%, 20%[ |
| Lamu | Hongwe | 4.07 (1.3, 12.3) | [2%, 10%[ | 1.88 (0.5, 6.6) | <2% |
| Lamu | Kiunga | 0 (0, 0) | <2% | 0 (0, 0) | <2% |
| Lamu | Mkomani | 0 (0, 0) | <2% | 27.78 (16.4, 43) | [20%, 50%[ |
| Lamu | Mkunubi | 14.14 (4.8, 34.7) | [10%, 50%[ | 29.32 (11.6, 56.7) | [20%, 50%[ |
| Lamu | Shella | 1 (0.3, 2.9) | <2% | 13.27 (2.6, 46.7) | [2%, 20%[ |
| Lamu | Witu | 20.22 (13.7, 28.9) | [10%, 50%[ | 19.02 (8.7, 36.8) | [2%, 20%[ |
| Mombasa | Airport | 1.56 (0.5, 4.8) | <2% | 0 (0, 0) | <2% |
| Mombasa | Bamburi | 1.06 (0.3, 3.3) | <2% | 1.06 (0.3, 3.4) | <2% |
| Mombasa | Bofu | 1.35 (0.6, 2.8) | <2% | 7.08 (4.1, 12.1) | [2%, 20%[ |
| Mombasa | Chaani | 0.86 (0.3, 2.6) | <2% | 3.42 (2, 5.7) | [2%, 20%[ |
| Mombasa | Changamwe | 4.4 (3.3, 5.8) | [2%, 10%[ | 1.96 (0.7, 5.2) | <2% |
| Mombasa | Free town | 0 (0, 0) | <2% | 3.26 (1.6, 6.6) | [2%, 20%[ |
| Mombasa | Jomvu Kuu | 0 (0, 0) | <2% | 0.87 (0.3, 2.4) | <2% |
| Mombasa | Junda | 2.88 (2.2, 3.7) | [2%, 10%[ | 4.31 (2.6, 7.2) | [2%, 20%[ |
| Mombasa | Kadzandani | 3.08 (1.6, 5.7) | [2%, 10%[ | 4 (1.1, 13.4) | [2%, 20%[ |
| Mombasa | Kipevu | 0 (0, 0) | <2% | 0.43 (0.1, 2.5) | <2% |
| Mombasa | Kongowea | 4.55 (2.2, 9.3) | [2%, 10%[ | 3.6 (1.5, 8.4) | [2%, 20%[ |
| Mombasa | Likoni | 2.74 (0.8, 9.4) | [2%, 10%[ | 1.36 (0.5, 3.7) | <2% |
| Mombasa | Magogoni | 0.97 (0.3, 3) | <2% | 1.9 (0.9, 4) | <2% |
| Mombasa | Majengo | 0 (0, 0) | <2% | 2.44 (0.9, 6.3) | [2%, 20%[ |
| Mombasa | Miritini | 0 (0, 0) | <2% | 1.38 (0.2, 7.3) | <2% |
| Mombasa | Mjambere | 4.91 (2, 11.8) | [2%, 10%[ | 5.68 (3.5, 9.2) | [2%, 20%[ |
| Mombasa | Mji wa Kale | 0.51 (0.1, 3) | <2% | 0.5 (0.1, 3) | <2% |
| Mombasa | Mkindani | 4.31 (2.2, 8.1) | [2%, 10%[ | 1.83 (0.7, 4.5) | <2% |
| Mombasa | Mkomani | 0.39 (0.1, 2.4) | <2% | 1.56 (0.7, 3.4) | <2% |
| Mombasa | Mtongwe | 0 (0, 0) | <2% | 13.18 (7.5, 22.3) | [2%, 20%[ |
| Mombasa | Mtopanga | 4.88 (2.4, 9.8) | [2%, 10%[ | 0.81 (0.3, 2.5) | <2% |
| Mombasa | Mwakirunge | 0.58 (0.1, 3.1) | <2% | 11.56 (5.7, 22) | [2%, 20%[ |
| Mombasa | Port Reitz | 0 (0, 0) | <2% | 3.29 (0.7, 13.9) | [2%, 20%[ |
| Mombasa | Shanzu | 4 (2, 7.9) | [2%, 10%[ | 17.33 (9.6, 29.3) | [2%, 20%[ |
| Mombasa | Shika Adabu | 3.63 (2.1, 6.3) | [2%, 10%[ | 4.46 (2.3, 8.6) | [2%, 20%[ |
| Mombasa | Shimanzi | 1.02 (0.3, 3.4) | <2% | 3.55 (2.2, 5.7) | [2%, 20%[ |
| Mombasa | Tibwani | 2.4 (0.9, 6.4) | [2%, 10%[ | 0 (0, 0) | <2% |
| Mombasa | Tononoka | 0.85 (0.3, 2.4) | <2% | 3.39 (1.4, 8) | [2%, 20%[ |
| Mombasa | Tudor | 0 (0, 0) | <2% | 2.92 (1.3, 6.6) | [2%, 20%[ |
| Mombasa | Ziwa La Ngombe | 2.16 (0.8, 5.8) | [2%, 10%[ | 3.28 (1.4, 7.3) | [2%, 20%[ |
| Taita Taveta | Bomeni | 1.24 (0.6, 2.6) | <2% | 0.81 (0.1, 4.6) | <2% |
| Taita Taveta | Bura | 0 (0, 0) | <2% | 0 (0, 0) | <2% |
| Taita Taveta | Chala | 4.09 (1.5, 10.5) | [2%, 10%[ | 2.26 (0.9, 5.8) | [2%, 20%[ |
| Taita Taveta | Chawia | 0 (0, 0) | <2% | 0 (0, 0) | <2% |
| Taita Taveta | Kaloleni | 1.13 (0.5, 2.8) | <2% | 1.12 (0.3, 3.6) | <2% |
| Taita Taveta | Kasigau | 0.52 (0.1, 3.3) | <2% | 0.51 (0.1, 2.6) | <2% |
| Taita Taveta | Mahoo | 13.86 (5.9, 29.1) | [10%, 50%[ | 1.69 (0.7, 3.8) | <2% |
| Taita Taveta | Marungu | 0.48 (0.1, 2.9) | <2% | 0 (0, 0) | <2% |
| Taita Taveta | Mata | 4.62 (3, 7.1) | [2%, 10%[ | 0.99 (0.4, 2.7) | <2% |
| Taita Taveta | Mboghoni | 10.98 (7.2, 16.4) | [10%, 50%[ | 1.92 (0.6, 6) | <2% |
| Taita Taveta | Mbololo | 0 (0, 0) | <2% | 0 (0, 0) | <2% |
| Taita Taveta | Mwanda | 1.06 (0.2, 5.6) | <2% | 1.57 (0.7, 3.3) | <2% |
| Taita Taveta | Mwatate | 0.43 (0.1, 2.4) | <2% | 0.83 (0.1, 4.7) | <2% |
| Taita Taveta | Ngolia | 14.49 (2, 58.1) | [10%, 50%[ | 0.47 (0.1, 2.5) | <2% |
| Taita Taveta | Rong'e | 0.4 (0.1, 2.3) | <2% | 0 (0, 0) | <2% |
| Taita Taveta | Sagala | 3.21 (1.6, 6.2) | [2%, 10%[ | 0 (0, 0) | <2% |
| Taita Taveta | Werugha | 0 (0, 0) | <2% | 0 (0, 0) | <2% |
| Taita Taveta | Wumingu | 0 (0, 0) | <2% | 1.71 (0.6, 5) | <2% |
| Taita Taveta | Wundanyi | 0.76 (0.1, 4.5) | <2% | 0.6 (0.1, 2.9) | <2% |
| Taita Taveta | Wusi | 0 (0, 0) | <2% | 0 (0, 0) | <2% |
| Tana River | Bangale | 0 (0, 0) | <2% | 2.02 (0.4, 10.4) | [2%, 20%[ |
| Tana River | Bura-hiramani | 37.4 (23.7, 53.5) | [10%, 50%[ | 3.51 (2.3, 5.3) | [2%, 20%[ |
| Tana River | Chewani | 25.99 (12.9, 45.4) | [10%, 50%[ | 1.31 (0.4, 4.2) | <2% |
| Tana River | Chewele | 8.53 (3, 21.9) | [2%, 10%[ | 1.87 (0.7, 5.1) | <2% |
| Tana River | Garsen Central | 18.34 (12.4, 26.4) | [10%, 50%[ | 15.81 (11.3, 21.7) | [2%, 20%[ |
| Tana River | Garsen North | 9.52 (5.1, 17.1) | [2%, 10%[ | 1.9 (0.3, 10.5) | <2% |
| Tana River | Garsen South | 29.59 (15.2, 49.6) | [10%, 50%[ | 0.58 (0.1, 2.9) | <2% |
| Tana River | Garsen West | 4.08 (0.8, 18.2) | [2%, 10%[ | 0.68 (0.1, 3.2) | <2% |
| Tana River | Kinakomba | 47.47 (33.3, 62) | [10%, 50%[ | 6.91 (4.1, 11.5) | [2%, 20%[ |
| Tana River | Kipini East | 31 (11.9, 59.8) | [10%, 50%[ | 14.93 (6.7, 30.1) | [2%, 20%[ |
| Tana River | Kipini West | 7.63 (2.7, 19.6) | [2%, 10%[ | 51.91 (41.8, 61.8) | >=50% |
| Tana River | Madogo | 4.55 (2.6, 7.7) | [2%, 10%[ | 0 (0, 0) | <2% |
| Tana River | Mikinduni | 27.36 (22.5, 32.8) | [10%, 50%[ | 5.29 (2.8, 9.7) | [2%, 20%[ |
| Tana River | Sala | 2.63 (0.5, 12.5) | [2%, 10%[ | 3.97 (2, 7.8) | [2%, 20%[ |
| Tana River | Wayu | 26.71 (16.5, 40.2) | [10%, 50%[ | 2.65 (2.3, 3.1) | [2%, 20%[ |
